# Placental Dysfunction Underlies Increased Risk of Fetal Growth Restriction and Stillbirth in Advanced Maternal Age Women

**DOI:** 10.1038/s41598-017-09814-w

**Published:** 2017-08-29

**Authors:** Samantha C. Lean, Alexander E. P. Heazell, Mark R. Dilworth, Tracey A. Mills, Rebecca L. Jones

**Affiliations:** 1 Maternal and Fetal Health Research Centre, Division of Developmental Biology and Medicine, University of Manchester, Manchester, United Kingdom; 2 St. Mary’s Hospital, Manchester Academic Health Science Centre, Central Manchester University Hospitals, NHS Foundation Trust, Manchester, United Kingdom

## Abstract

Pregnancies in women of advanced maternal age (AMA) are susceptible to fetal growth restriction (FGR) and stillbirth. We hypothesised that maternal ageing is associated with utero-placental dysfunction, predisposing to adverse fetal outcomes. Women of AMA (≥35 years) and young controls (20–30 years) with uncomplicated pregnancies were studied. Placentas from AMA women exhibited increased syncytial nuclear aggregates and decreased proliferation, and had increased amino acid transporter activity. Chorionic plate and myometrial artery relaxation was increased compared to controls. AMA was associated with lower maternal serum PAPP-A and sFlt and a higher PlGF:sFlt ratio. AMA mice (38–41 weeks) at E17.5 had fewer pups, more late fetal deaths, reduced fetal weight, increased placental weight and reduced fetal:placental weight ratio compared to 8–12 week controls. Maternofetal clearance of ^14^C-MeAIB and ^3^H-taurine was reduced and uterine arteries showed increased relaxation. These studies identify reduced placental efficiency and altered placental function with AMA in women, with evidence of placental adaptations in normal pregnancies. The AMA mouse model complements the human studies, demonstrating high rates of adverse fetal outcomes and commonalities in placental phenotype. These findings highlight placental dysfunction as a potential mechanism for susceptibility to FGR and stillbirth with AMA.

## Introduction

Advanced maternal age (AMA; ≥35 years of age) is increasingly prevalent in more economically developed countries, with AMA mothers contributing 20% and 16% of the obstetric population in the UK and USA respectively in 2014^1, 2^. AMA has established associations with adverse pregnancy outcomes, particularly those resulting from placental dysfunction, including fetal growth restriction (FGR), stillbirth and preeclampsia (PE)^3–6^. However, the pathological mechanisms underlying this increased susceptibility are not known. This increased risk is not only independent of maternal co-morbidities^7^, but also despite displaying factors that favour healthy pregnancy outcomes, such as higher socio-economic status, pre-conceptional nutritional supplementation, non-smoking and high attendance to antenatal care^8–10^. Therefore, AMA is an independent risk factor for poor pregnancy outcome.

Placental dysfunction is a major cause of FGR and placental causes are identified in up to 65% of stillbirths^11, 12^. Gross placental morphology is altered in both conditions, including lower placental weight^13, 14^, and irregular placental shape and umbilical insertion^15^. Placentas from pregnancies affected by FGR are poorly vascularised, with reduced vascular branching and fewer capillaries^16–18^. Increased apoptosis^19, 20^, decreased proliferation^16^ and increased numbers of syncytial nuclear aggregates (SNAs)^21, 22^ are also found in FGR placentas. These structural abnormalities are even more pronounced in stillbirths associated with FGR, and are commonly detected in stillbirths of otherwise unknown cause^11^.

Abnormal placental function is also apparent in FGR and stillbirths. Reduced placental amino acid transport has been reported in FGR^23, 24^, including a reduction in amino acid transporters system A^25^ and TauT activity^26^. FGR is also associated with impaired placental endocrine function, with lower maternal serum concentrations of pregnancy associated plasma protein (PAPP)-A^27^, placental lactogen (hPL)^28^ and placental growth factor (PlGF)^29^. Clinical measures of placental dysfunction, including abnormal Doppler ultrasound resistance indices in both umbilical and uterine arteries (indicating increased vascular resistance and impaired spiral artery adaptations) are more prevalent in FGR and stillbirth^30, 31^. Consistent with these clinical observations, chorionic plate resistance arteries in FGR exhibit reduced constriction and exacerbated relaxation^32^ as assessed by wire myography. Conversely, myometrial arteries show attenuated relaxation in FGR^33^. Whether any of these aspects of placental development, structure or function are altered in mothers of AMA and whether they underpin the susceptibility to FGR and stillbirth is unknown.

Animal models are frequently used to dissect the role of placental dysfunction in adverse pregnancy outcomes. A previous study found decreased pup viability in ageing (40 week old) mice compared to younger controls, coupled with histological evidence of stunted uterine artery remodelling^34^. Although a recent study using aged pregnant rats found altered maternal uterine vascular function^35^, placental function analyses were not conducted in either of these rodent models.

The current study addressed the hypothesis that AMA is associated with placental and utero-placental dysfunction. Using a broad array of investigations in both human pregnancies and a murine model of AMA we identified multiple features of placental and utero-placental dysfunction, providing a potential mechanistic link for the increased FGR and stillbirth rates with AMA.

## Results

### Participant Demographics

Table 1 shows the demographics and obstetric outcome for the total study population. Demographic and biophysical characteristics were matched between maternal age groups wherever possible. However, paternal age was significantly higher for mothers of AMA compared to controls (*p* < 0.0001). More women of AMA were home owners (*p* < 0.05) and a greater proportion were parous (*p* < 0.05) but not grandparous. No differences were seen in IMD (a proxy for socioeconomic status), although there may potentially be a negative linear relationship if a continuum of maternal ages had been included. Although mothers ≥40 years had higher rates of previous miscarriage (*p* < 0.05), there were no differences in incidence of previous adverse pregnancy outcome (stillbirth or SGA infant <10^th^ individualised birthweight centile (IBC)) between age groups. No participants in this cohort had stated use of assistive reproductive techniques.

**Table 1:** Demographic and obstetric outcome data for total study population.

Demographics/Outcome	20–30 Years (n = 68)	35–39 Years (n = 47)	≥40 Years (n = 58)	*p* value
**Maternal Age** Years	**26** (20–30)	**37** (35–39)***	**42** (40–48)***	**<0.0001** ^**a**^
*Paternal Age Years	**29** (21–39)	**36** (27–45)***	**43** (25–55)***	**<0.0001** ^**a**^
**Ethnicity**				
*European*	**91.2%** (62)	**97.5%** (39)	**87.9% (**51)	NS
*Other*	**8.8%** (4)	**2.5%** (1)	**12.1%** (6)	NS
**BMI** kg/m^2^	**23.6** (19.0–29.9)	**24.0** (18.5–28.6)	**24.6** (19.0–29.6)	NS
**Marital Status**				
*Married*	**50.0%** (50)	**70.0%** (28)	**70.0%** (28)	NS
*Partner*	**47.5%** (19)	**30.0%** (12)	**25.0%** (10)	NS
*Single*	**2.5%** (1)	**0.0%** (0)	**2.5%** (1)	NS
*Employment				
*Employed*	**90.0%** (36)	**77.5%** (31)	**77.5%** (31)	NS
**Smoking Status**				
*Non-Smokers*	**100%** (68)	**100%** (47)	**100%** (58)	NS
*Housing Status				
*Owns*	**57.4%** (23)	90.0%* (36)	**95.0**%* (38)	**<0.05** ^**b**^
*IMD Score	**18.9** (2.28–72.0)	**16.1** (1.9–76.1)	**15.5** (1.9–73.5)	**NS**
**Parity**				
*Primiparous*	**47.5%** (19)	20.0%* (9)	22.4%* (13)	**<0.05** ^**b**^
*Parous*	**52.5%** (21)	80.0%* (32)	77.6%* (45)	**<0.05** ^**b**^
*Grandparous*	**0.0%** (0)	**2.5%** (1)	**5.2%** (3)	NS
*Previous Miscarriage	**25.0%** (11)	**35.0%** (14)	52.5%* (21)	**<0.05** ^**b**^
*Previous APO				
*% of parous women*	**14.3%** (3)	**25.0%** (8)	**22.6%** (7)	NS
*Previous/Current Fertility Treatment	**0.0%** (0)	**0.0%** (0)	**0.0%** (0)	NS
**Gestation at Delivery**				
*Weeks* + *Days*	**40 + 2** (37 + 6–42 + 2)	**39 + 5** (37 + 4–42 + 1)*	**39 + 2** (37 + 1–42 + 4)*	**<0.01** ^a^
**Birthweight** (g)	**3380** (2620–4300)	**3500** (2780–4100)	**3481** (2840–4420)	NS
**IBC**	**45.9** (11.0–94.4)	**40.1** (10.4–91.5)	**46.9** (11.1–89.1)	NS
**IOL**	**17.6%** (12)	**17.5%** (7)	**35.9%** (15)	NS
**Mode of Delivery**				
*NVD*	**33.8%** (23)	**65.0%** (26)	**39.7%** (23)	NS
*ELCS*	**48.5%** (33)	22.5%** (9)	**43.1%** (25)	<**0.01** ^b^
*EMCS*	**4.5%** (3)	**5.0%** (2)	10.3%* (6)	**<0.05** ^b^
*INST*	**13.2%** (9)	7.5%* (3)	6.9%* (4)	**<0.05** ^b^
**Male Infant**	**50.0%** (34)	**40.0%** (16)	**56.8%** (33)	NS
**CAPO**	**0.0%** (0)	**0.0%** (0)	**0.0%** (0)	NS

Data as median (range) or percentage (number). *p < 0.05, **p < 0.01, ***p < 0.001 compared to control group (20–30 years). ^a^Kruskal-Wallis with Dunn’s multiple comparisons. ^b^Fisher’s exact probability test. BMI = Body Mass Index; IMD = Index of Multiple Deprivation; APO = Adverse Pregnancy Outcome, IBC = Individualised Birthweight Centile; IOL = Induction of Labour; NVD = Normal Vaginal Delivery; ELCS = Elective Caesarean Section; EMCS = Emergency Caesarean Section; INST = Instrumental Delivery; CAPO = Composite Adverse Pregnancy Outcome. *Data only available from a sub-cohort (20–30 years n = 40; 35–39 years n = 47; ≥40 years n = 40.

By study design, all women had uncomplicated pregnancies defined as: live birth, delivery between 37–42 weeks gestation, birthweight >2500 g, IBC between 10–95^th^ centile and absence of pre-existing or pregnancy-related maternal disease. Mothers of AMA delivered on average 5–7 days earlier than 20–30 year olds (*p* < 0.01), but no differences were seen in birthweight or IBC. Rates of induction of labour and normal vaginal delivery (NVD) were not different between groups. However, AMA groups had higher emergency Caesarean section and lower instrumental vaginal delivery rates (*p* < 0.05).

### Human Placental Analyses

Placental analyses were performed on matched sub-cohorts, the participants of which did not differ in demographic or obstetric outcomes from the total study population unless stated otherwise.

### Placental Weight and Morphology

**Figure 1:**
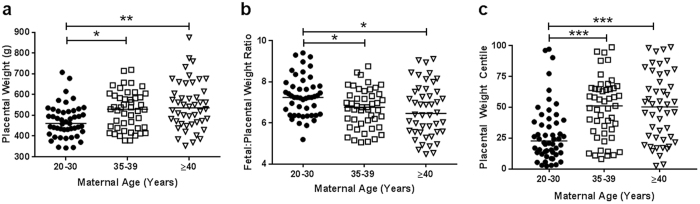
Effect of advanced maternal age on human placental weights. (**a**) Trimmed placental weight, (**b**) Fetal:Placental Weight ratio and (**c**) placental centiles. Data presented as median, Kruskal-Wallis with Dunn’s posthoc test, *p < 0.05, **p < 0.01, ***p < 0.005. (20–30 (n = 47), 35-39 (n = 47) and ≥40 (n = 46) years of age).

Placental trimmed weights were taken post-delivery from a subgroup of participants (Fig. 1; 20–30 (n = 47), 35–39 (n = 47) and ≥40 (n = 46) years). Despite there being no differences in birthweight (Table 1), placental weights were higher in both 35–39 year (*p* < 0.05) and ≥40 year old AMA pregnancies (*p* < 0.01; Fig. 1a). Placental centiles increased in both mothers 35–39 and ≥40 years compared to young controls (*p* < 0.005, Fig. 1c). Fetal:placental weight ratios (FW:PW) were reduced in mothers aged 35–39 and ≥40 years compared to 20–30 years (*p* < 0.01; Fig. 1b).

**Figure 2:**
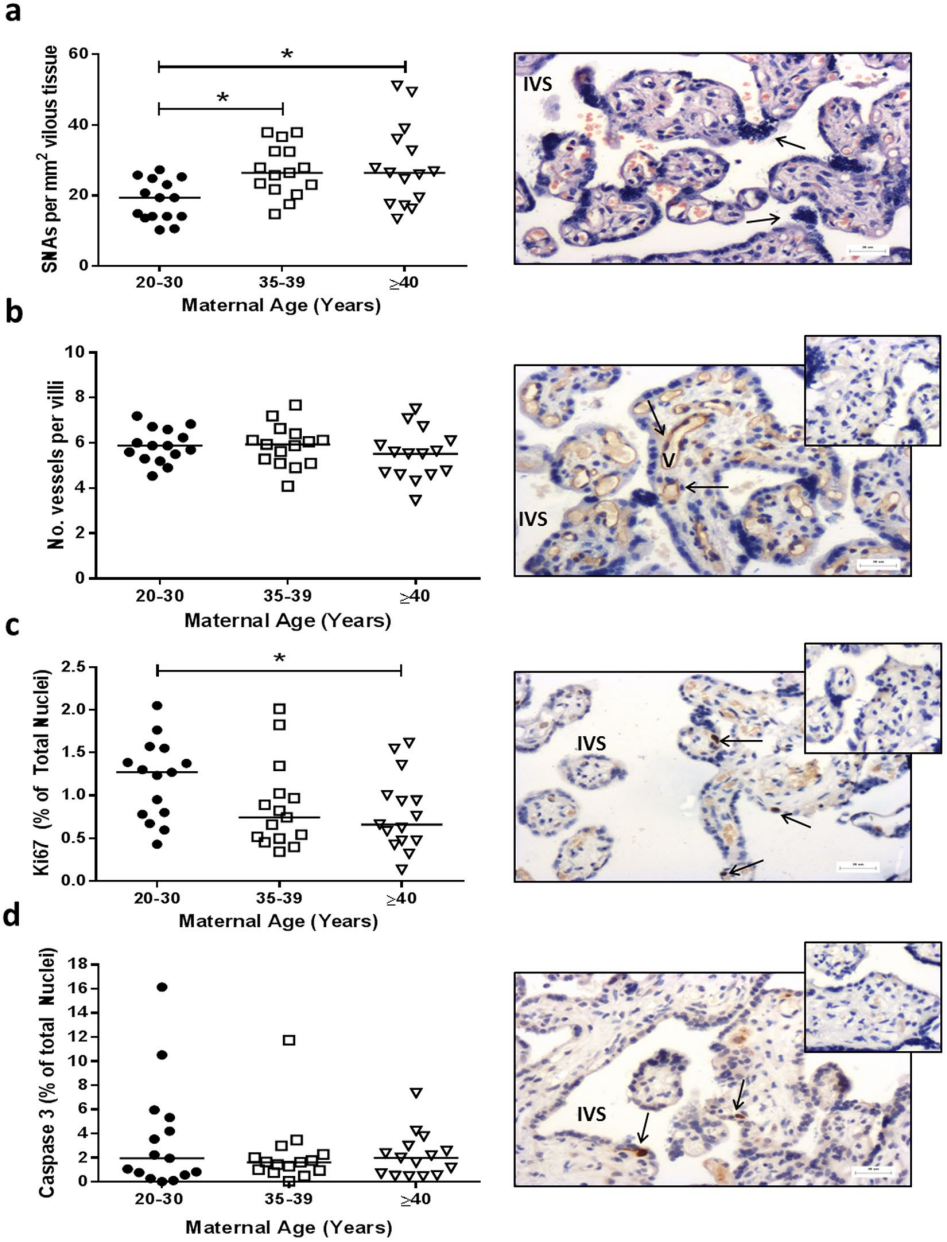
Effect of advanced maternal age on human placental morphology and turnover. (**a**) Syncytial nuclear aggregates (SNAs) following haematoxylin and eosin staining, (**b**) Number of vascular cross sections per terminal villi (following CD31 immunostaining to identify endothelial cells (Image), (**c**) Proliferative index (based on ki67 immunostaining) and (**d**) Apoptotic index (based on Caspase 3 immunostaining). Insets show negative controls. Kruskal-Wallis with Dunn’s posthoc test, *p < 0.05 (n = 14–15/group). Representative images are shown for each histological or immunohistochemical stain. STB = syncytiotrophoblast; V = vessel; IVS = inter-villous space. Arrows indicate SNA (image **a**) or positive stain (images **b**–**d**).

Placentas used for histology were matched for gestational age, maternal BMI and were all from non-smokers (Supplementary Table 1). Mothers ≥40 years were less likely to have delivered by NVD (20.0% vs 62.5%; *p* = 0.05). There were higher numbers of SNAs in placentas from 35–39 and ≥40 year olds compared to 20–30 years olds (*p* < 0.05; Fig. 2a). There was no difference in number of capillaries per terminal villi (Fig. 2b) or number of avascular villi (not shown) with maternal age. Although apoptotic index was unaltered (Fig. 2d), reduced proliferative index in placentas from ≥40 year olds compared to controls was detected (*p* < 0.05; Fig. 2c).

### Placental Function

#### Placental amino acid uptake via system A and TauT

**Figure 3:**
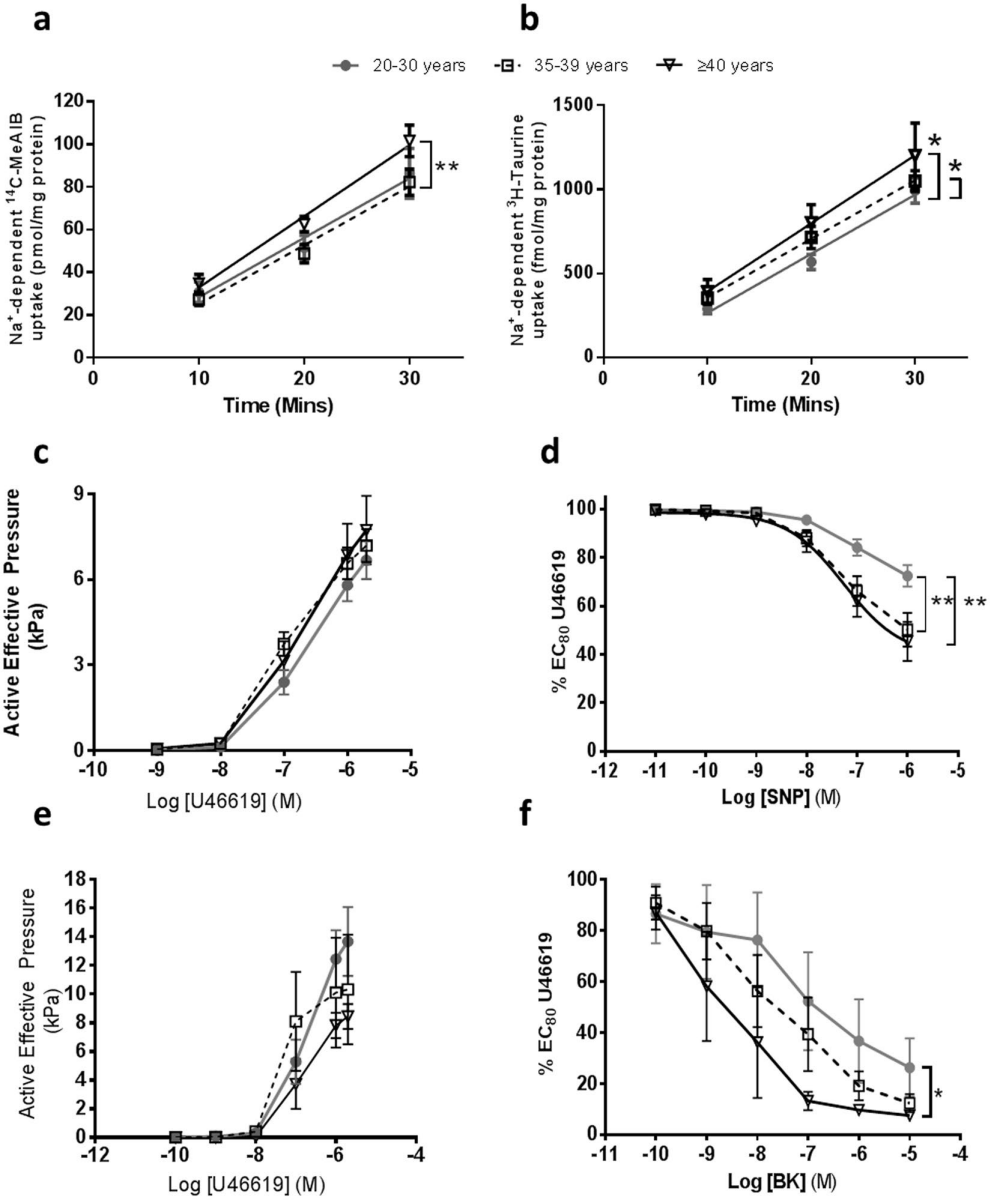
Effect of advanced maternal age placental and arterial function. (**a**) Placental system A activity (Na^+^-dependent ^14^C-MeAIB uptake), (**b**) placental TauT activity (Na^+^-dependent ^3^H-TauT uptake). Mean ± SEM; linear regression (difference in intercept and elevation, *p < 0.05, **p < 0.005). (**c**) Chorionic plate artery constriction in response to U46619 and (**d**) relaxation in response to sodium nitroprusside (SNP) in pre-constricted CPA (n = 15/age group). (**e**) Myometrial artery constriction to U46619, and (**f**) relaxation to bradykinin (BK) in myometrial vessels from women ages 20–30 (n = 9), 35–39 (n = 17) and ≥40 (n = 6) years of age. Mean ± SEM, 2-Way ANOVA with Bonferroni’s post hoc test, *p < 0.05, **p < 0.01.

The demographics for participants whose placentas were used for nutrient transport studies were matched to those in Table 1 (Supplementary Table 2) with a few variances. There was no difference in gestation at delivery or parity with maternal age. Although birthweight was not different between the maternal age groups, IBC was significantly higher in mothers ≥40 years compared to controls (*p* < 0.05). System A activity (Na^+^ dependent ^14^C-MeAIB uptake) was increased in placentas from mothers aged ≥40 years compared to those aged 35–39 years (*p* < 0.005, Fig. 3a). TauT activity (Na^+^ dependent ^3^H-Taurine uptake) was increased in placentas from both mothers aged 35–39 and ≥40 years compared to 20–30 year old counterparts (*p* < 0.05, Fig. 3b).

### Circulating Placental Hormones

**Figure 4:**
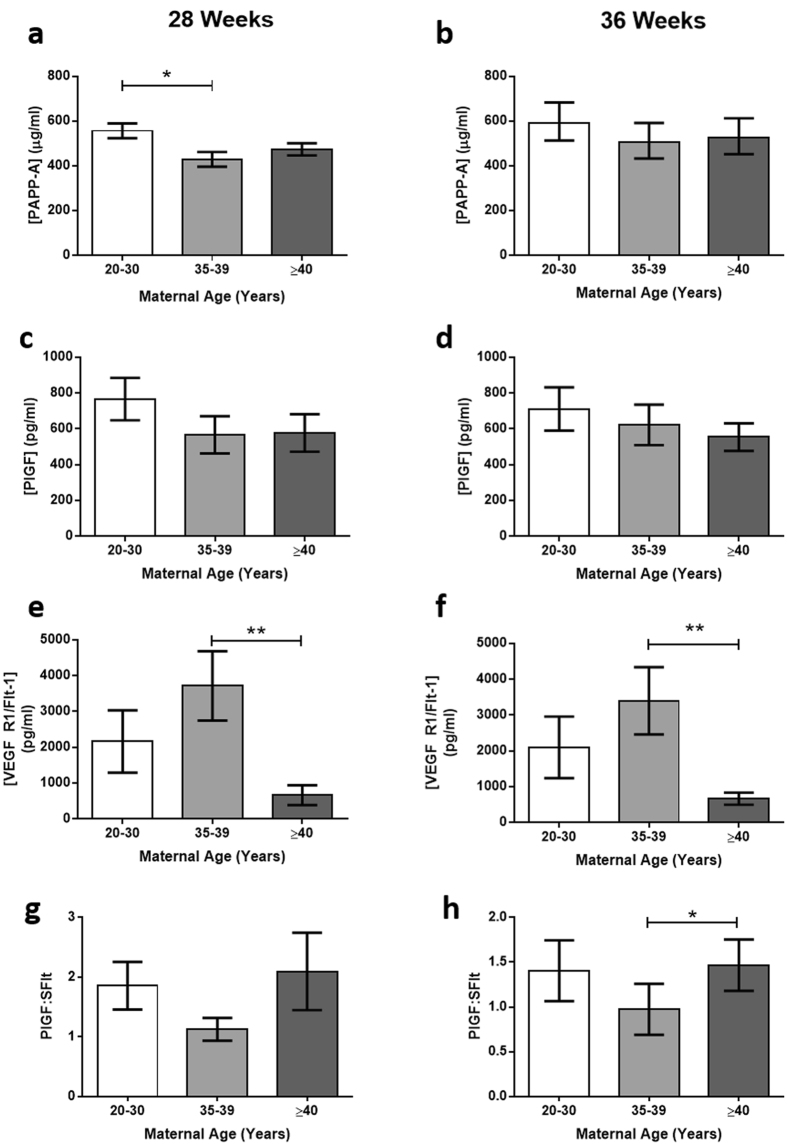
Effect of advanced maternal age on concentrations of maternal serum placental hormones at 28 and 36 weeks gestation. (**a**,**b**) Pregnancy associated plasma protein (PAPP)-A, (**c**,**d**) placental growth factor (PlGF), (**e**,**f**) soluble fms like tyrosine kinase-1 (VEGF R1/Flt-1; sFlt), G-H) PlGF:sFlt ratio. Mean ± SEM. ANOVA with Dunn’s multiple comparison test, *p < 0.05, **p < 0.01.

Circulating placental hormones were measured in maternal serum at 28 and 36 weeks gestation. Serum concentrations of PAPP-A at 28 weeks were decreased in women aged 35–39 years (*p* < 0.05), with a similar trend at 36 weeks (Fig. 4a, n = 40/group). No differences were found between maternal levels of hCG, progesterone, or hPL or PlGF between the age groups at either gestation (not shown). sFlt concentrations were lower in mothers ≥40 years compared to 35–39 year olds at 28 and 36 weeks gestation (*p* < 0.005 and *p* < 0.05, Fig. 4e,f). The PlGF:sFlt ratio (indicative of free PlGF) was increased in mothers ≥40 years of age compared to 35–39 year olds at 36 weeks gestation (*p* < 0.05, Fig. 4h).

### Chorionic Plate Artery (CPA) Wire Myography

Maternal age did not affect constriction of CPAs to U46619 (20–30 n = 15; 35–39 n = 16, ≥40 n = 15; Fig. 3c). When treated with increasing concentrations of the nitric oxide donor SNP, pre-constricted CPAs exhibited increased relaxatory response from women in both AMA groups compared to controls (*p* < 0.005, Fig. 3d).

### Myometrial Artery Wire Myography

Constriction of myometrial arteries to U46619 was variable but not affected by maternal age (n = 15/group; Fig. 3e). However, pre-constricted myometrial arteries from women ≥40 years old showed greater relaxation response to bradykinin than controls (*p* < 0.05; Fig. 3f).

### Mouse Model of AMA

#### Maternal Weight and Food Consumption throughout Ageing

Mice were monitored for maternal weight and food consumption throughout the ageing process. AMA mice gained on average 0.48 ± 0.15 g of maternal weight per week of ageing (median ± STDEV; n = 12) between 12–36 weeks of age. AMA mice weighed on average 7.6 g more than young control mice at E0.5 (30.35 ± 4.36 g vs 22.30 ± 1.68 g respectively; *p* < 0.0001 Mann-Whitney U, n = 25/group). Food consumption monitoring revealed that ageing dams consume decreasing quantities of food (3.4 ± 0.25 at 14 weeks, 3.2 ± 0.40 g at 27 weeks and 3.05 ± 0.37 g at 35 weeks g/day) from 14 to 27 (*p* < 0.05) and 14 to 35 weeks of ageing (*p* < 0.01, Kruskal Wallis, n = 8/group).

## Fetal and Placental Measurements

Litters were compared between control and AMA mice at E17.5. Data are displayed as litter averages (N) and total number of pups (n). AMA mice had 54% fewer total and viable pup numbers (*p* < 0.0001), twice as many resorptions (*p* < 0.0001) and more late fetal deaths (*p* < 0.01; control N = 34 (n = 156), AMA N = 23 (n = 99); Table 2). All the late fetal losses (non-viable pups) were in AMA mice, affecting 31% of AMA litters. The total number of conceptuses evident at E17.5 (viable/non-viable pups and reabsorptions) was lower in AMA than in young mice (*p* < 0.05; Table 2).

**Table 2:** Effect of advanced maternal age on pregnancy outcome in mice.

Measure	Control	AMA	AMA (NV)
Total Litter Size	**7** (4–12)	**4** (0–7)***	—
Viable Litter Size	**7** (4–12)	**4** (0–7)***	—
Resorptions	**0.5** (0–4)	**2** (0–7)***	—
Total Conceptuses litter size+resorptions	**8** (6–12)	**7** (1–10)***	—
Late Fetal Deaths	**0** (0–0)	**0** (0–2)***	—
Fetal Weight mg	**856** (778–938)	658*** (448–829)	435*** ^**†**^ (185–727)
Placental Weight mg	**82** (66–99)	101** (81–144)	**87** ^**†**^ (59–124)
FW:PW Ratio	**10.7** (8.8–12.1)	6.7*** (4.4–9.4)	4.2*** ^**†**^ (2.3–6.5)
Crown Rump mm	**27.7** (26.6–29.3)	25.1*** (19.7–28.0)	20.5*** (15.0–25.0)
Abdominal Circumference mm	**23.1** (21.3–24.9)	20.7*** (18.0–24.0)	17.0*** (14.0–20.0)
Head Circumference mm	**22.9** (21.5–24.6)	21.0** (18.0–24.0)	19.0** (14.0–26.0)
Head:Abdominal Circumference	**1.00** (0.88–1.09)	**1.03** (0.83–1.16)	**1.06** ^**†**^ (0.95–1.30)

Data are median (range). **p < 0.01, ***p < 0.001 compared to control group, ^†^p < 0.05 differences from AMA. Mann-Whitney test (for total and viable litter size, resorptions, conceptuses and late fetal deaths) or Kruskal-Wallis with Dunn’s multiple comparisons. FW:PW = Fetal:placental weight ratio; NV = Non-Viable.

**Figure 5:**
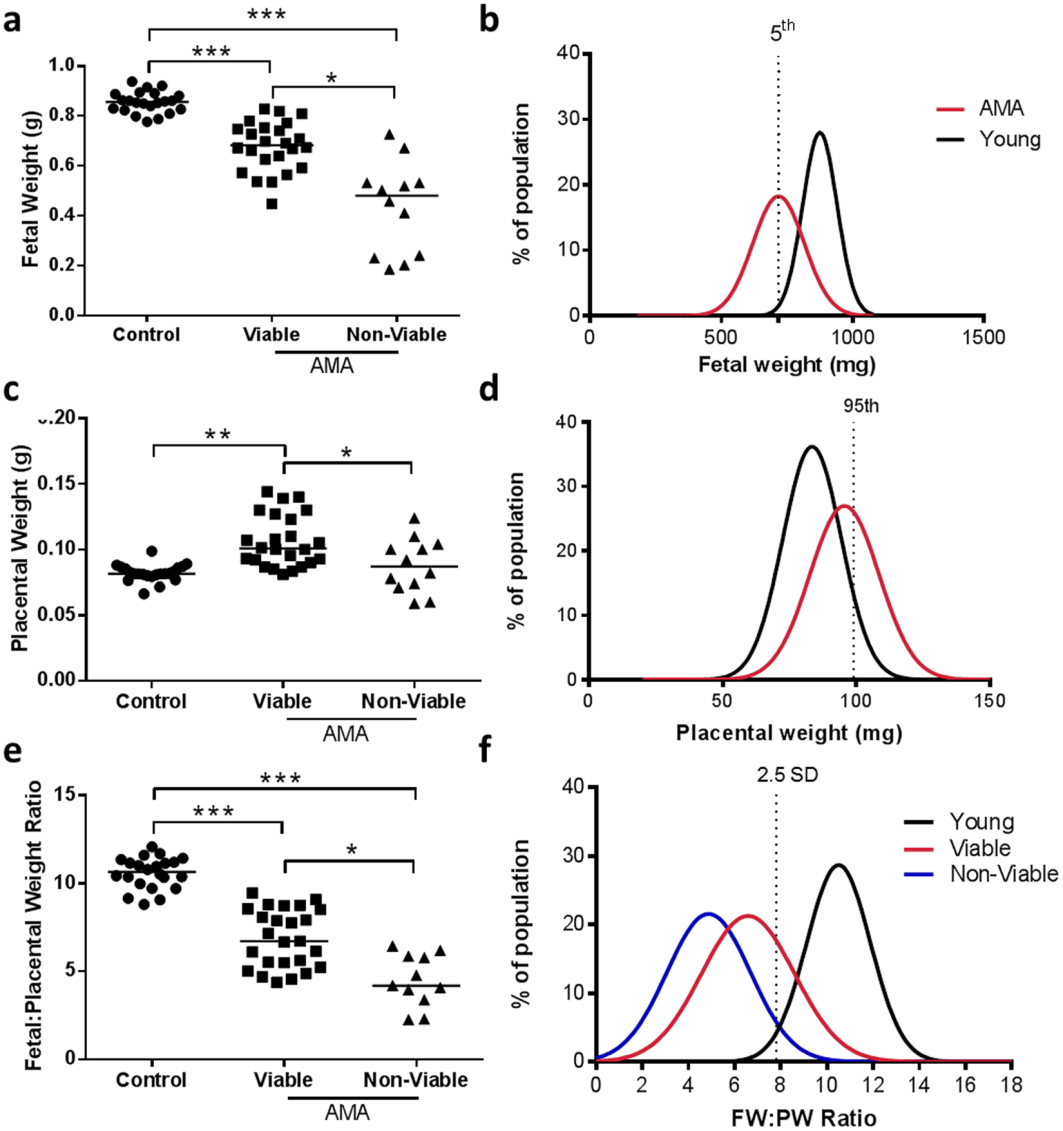
Fetal and placental measurements from control and advanced maternal age (AMA) mouse pregnancies. (**a**) Fetal weights, (**b**) fetal weight frequency distribution, (**c**) placental weights, (**d**) placenta weight frequency distribution, (**e**) fetal:placental weight ratios and (**f**) fetal:placental weight ratios frequency distribution. Data in (**a**,**c** and **e**) are presented as litter averages [N = 22 for controls (n = 159 individual fetuses), N = 13 for AMA viable (n = 99 individual fetuses), N = 12 for AMA non-viable (n = 14 individual fetuses)]. *p < 0.05, **p < 0.01, ***p < 0.001, Kruskal-Wallis with Dunn’s Multiple Comparison Test. For fetal weight frequency distribution (**b**) (control = black line, AMA = red line), the dotted line refers to 5^th^ centile of control population. For placental weight frequency distribution (**d**) the dotted line refers to 95^th^ centile of control population. For FW:PW ratios frequency distribution (**f**) (control = black line, AMA Viable = red line, AMA Non-Viable = blue line) the dotted line refers or 2.5 standard deviations (SD) away from the mean of control population.

Fetal and placental measurement data from AMA mice were analysed for viable and non-viable pups separately (control N = 22 (n = 165), AMA viable N = 13 (n = 86), AMA non-viable N = 12 (n = 15)). AMA viable fetal weights were lower than controls and AMA non-viable pups weighed less than both control and AMA viable pups (by 21% and 49% respectively *p* < 0.0001; Fig. 5a).

When analysed as a frequency distribution, the fetal weight curve from AMA pregnancies was shifted to the left compared with the control group, with 56% of AMA fetuses weighing < 5^th^ centile of the control population (Fig. 5b). AMA viable-pups had higher placental weights compared to control (*p* < 0.005). However, placentas of AMA non-viable pups weighed significantly less than the viable AMA group, but were comparable to controls (*p* < 0.005; Table 2, Fig. 5c). AMA placental weights showed a rightward shift in weight frequency distribution compared to control, with 48% of AMA placentas measuring >95^th^ centile of the control population (Fig. 5d). FW:PW ratios were lower in AMA viable-pups compared to controls and lower still in AMA non-viable pups compared to both control and AMA viable (p < 0.0001; Fig. 5e). Furthermore, there was a leftward shift in frequency distribution, with 71% and 92% of AMA viable and non-viable pups respectively falling more than 2.5 standard deviations from the mean of the control population (Fig. 5f).

All anthropometric measures (crown rump, abdominal and head circumference) were reduced in AMA mice and this was exacerbated if the pup was non-viable at E17.5 (*p* < 0.0001; Table 2). Head:abdominal circumference ratio was decreased between non-viable AMA pups and controls, indicating head-sparing in these offspring (*p* < 0.05; Table 2).

### Placental Function

Fetuses that were determined to be non-viable at E17.5 and their placentas were excluded from functional analyses.

### System A and TauT Maternofetal Clearance

**Figure 6:**
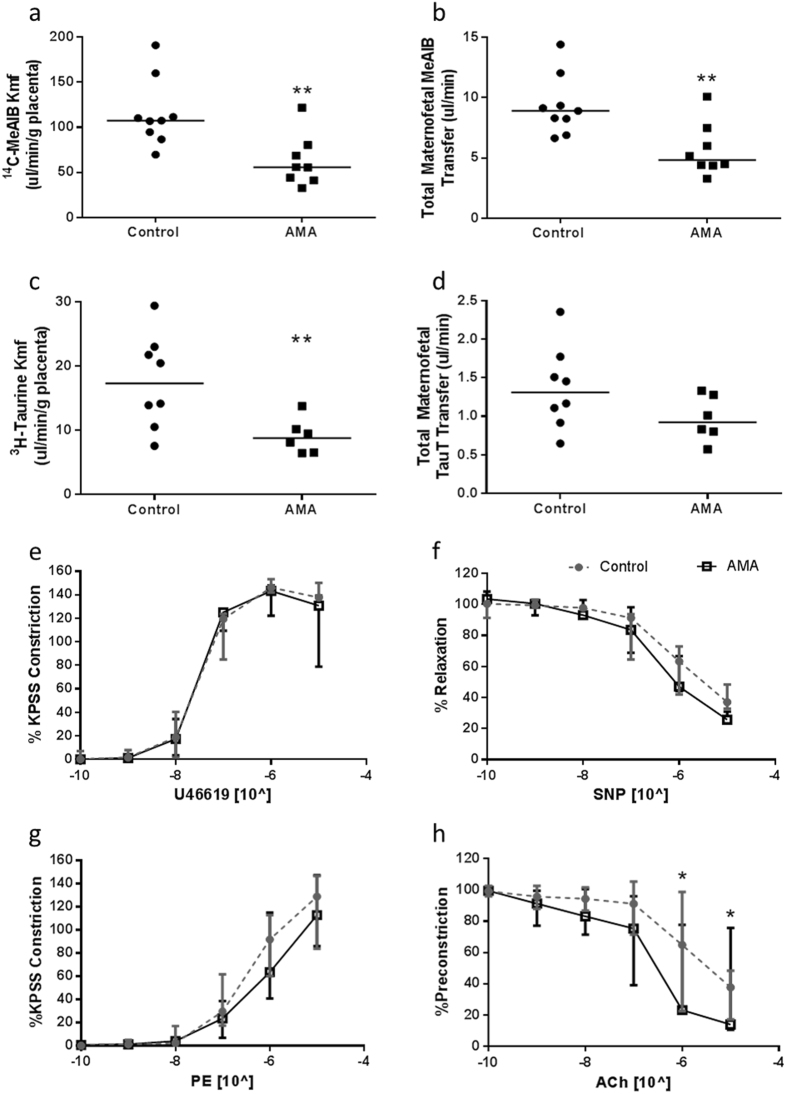
Effect of maternal age on AMA murine amino acid transfer and umbilical and uterine artery function. (**a**) Rate of uni-directional maternofetal clearance of ^14^C-MeAIB per gram of placenta (µl/min/g placenta), (**b**) total maternofetal transfer of ^14^C-MeAIB independent of fetal or placental size, (**c**) the rate of uni-directional maternofetal clearance of ^3^H-Taurine per gram of placenta (µl/min/g placenta), and (**d**) total maternofetal transfer of ^3^H-Taurine independent of fetal or placental size **p < 0.001, Mann-Whitey U Test. Effect of advanced maternal age on umbilical artery: (**e**) constriction to U46619, and (**f**) relaxation to sodium nitroprusside (SNP) (control n = 6, AMA n = 4) and uterine artery: (**g**) constriction to a phenylephrine and (**h**) relaxation to acetyl choline (ACh). Data are mean ± SEM. 2 Way ANOVAs with Bonferroni’s Multiple Comparison (con n = 7, AMA n = 5), *p < 0.05.

The rate of uni-directional maternofetal clearance of ^14^C-MeAIB per gram of placenta was reduced in AMA mice (*p* < 0.005; control N = 9 (n = 66), AMA N = 8 (n = 37); Fig. 6a) compared to controls. Total ^14^C-MeAIB transfer, independent of placental size, was also reduced (Fig. 6b). Uni-directional maternofetal clearance of ^3^H-Taurine was also reduced in AMA mice (*p* < 0.01; control N = 8 (n = 63), AMA N = 6 (n = 26); Fig. 6c) compared to controls with correction for placental weight. Total maternofetal transfer of ^3^H-Taurine, independent of placental size, showed no differences between groups (Fig. 6d).

### Uterine and Umbilical Artery Myography

Umbilical arteries exhibited no differences in constriction responses to U46619 or relaxation to SNP between maternal age groups (control N = 6 (n = 24), AMA N = 4 (n = 13), Fig. 6e,f). Uterine arteries from AMA dams showed no differences in constriction in response to PE compared to controls (Fig. 6g), but pre-constricted arteries exhibited increased relaxation to ACh when compared to controls (*p* < 0.001; control N = 7, AMA N = 5, Fig. 6h).

## Discussion

This study addressed the hypothesis that utero-placental dysfunction is an underlying mechanism for susceptibility to FGR and stillbirth in AMA pregnancies. Detailed characterisation studies revealed increased placental weight, but reduced placental efficiency, in pregnancies from women of AMA, together with alterations in placental morphology and function resembling the placental phenotype in FGR and stillborn pregnancies. This phenotype was evident despite apparently normal pregnancy outcomes, indicating adverse effects of maternal ageing on the placenta and potential adaptive responses to achieve a normal birthweight. The abnormal placental phenotype was more marked in women ≥40 years of age, consistent with a higher risk of adverse pregnancy outcomes than women aged <35 years^36^. To exclude confounding effects in clinical studies, we developed a mouse model of AMA. AMA mice experienced a more severe pregnancy phenotype than in the human pregnancies, with reduced fecundity, increased resorptions and late fetal deaths, and over half of the offspring were classed as FGR (fetal weight <5^th^ centile). Many similarities were detected in the placental phenotype in the mouse model and human pregnancies, particularly increased placental weight, decreased placental efficiency and altered uteroplacental vascular function, although many of the changes were exaggerated consistent with the more severe fetal outcomes detected. These complementary data from human and murine studies support the hypothesis that maternal ageing is associated with placental dysfunction, which may contribute to the higher rates of stillbirth and FGR in AMA pregnancies.

Morphological differences were apparent in the placentas from women of AMA compared to younger counterparts from gestationally matched, uncomplicated pregnancies, including increased numbers of SNAs and reduced rates of trophoblast proliferation. Both are features of placental dysfunction in pathological pregnancies, including FGR, stillbirth and pre-eclampsia^37, 38^, but also of post-term pregnancies signifying placental ageing^39^. Placental ageing is a probable cause of the dramatic increased risk of stillbirth in post term pregnancies (>41 weeks gestation)^3, 6, 40^. Accelerated placental ageing is a potential explanation for a comparable risk of stillbirth in women ≥40 years at 39 weeks gestation^40^. Further mechanistic studies are required to investigate this hypothesis further.

Reduced placental efficiency, indicated by a lower FW:PW ratio^41^, occurs in FGR pregnancies^42^. In women ≥40 years with uncomplicated pregnancies, placental weight was higher than in controls. This was accompanied by changes in placental function – namely elevated amino acid transport and augmented relaxation of CPA and myometrial arteries in response to vasodilators – which together would be proposed to be beneficial for fetal growth and survival. The absence of a parallel increase in birthweight is indicative of either deficiencies in other important aspects of placental function, or an adverse maternal environment that is unfavourable to fetal growth. In the context of this AMA environment, there are placental changes in terms of increased size and altered function. Whether these changes are an adaptation by the placenta in order to maintain appropriate growth of the fetus, or simply a consequence of the altered maternal environment, requires further study. These studies provide supportive evidence for the concept of placental adaptations in human pregnancies, as is well established in murine pregnancies^43^ (e.g. *Igf2*, placental specific *Igf2* and 11-βHSD2 knock out models), where placental amino acid transport is enhanced to compensate for reduced placental size. These support normal fetal growth and failure of the adaptations results in FGR^44^. Such functional adaptions may be driven by fetal nutrient demand, as in the mouse^43, 45^, or in response to an adverse environment, for example elevated oxidative stress, as reported in neurones^46, 47^, or as a consequence of the ageing maternal environment.

The measurement of third trimester placental hormones in maternal circulation has been previously studied to assess placental function and risk of adverse pregnancy outcome^28, 48^. Lower maternal serum PAPP-A in early gestation is associated with PE, prematurity, FGR and stillbirth^27, 49^, and third trimester concentrations of hPL and PlGF are decreased in FGR^28, 29, 50^. Lower levels of placental hormones are presumed to infer reduced placental size and/or function in FGR^28, 49, 51^. Although PlGF concentrations were not altered, the higher PlGF:SFlt ratio indicates higher free bioavailable PlGF, which is consistent with the increased placental size detected. The absence of alterations in hCG, hPL or progesterone concentrations, and the significantly lower PAPP-A, in AMA pregnancies are thus prominent findings given the higher placental weight and instead imply reduced placental endocrine function.

The human studies were complemented by studies of a mouse model of AMA, which showed similarities in pregnancy outcomes to those reported by large epidemiological studies of human AMA pregnancies, including reduced fecundity^52^, increased early fetal loss^53^, FGR^10^ and increased late stillbirths^54^. These were detected at higher frequency than in human pregnancies, indicating a more extreme model, which is valuable in terms of delineating the mechanisms causing stillbirth with AMA. 40 week old mice were chosen as this best represents the age range of women in the human studies^34, 55^. Similarities were detected in the placental phenotype in both mouse and human studies, most notably increased placental weight and reduced PW:FW. The increase in placental weight was only apparent in viable pups at E17.5, supporting the hypothesis from the human studies for increased placental growth as an adaptation to promote fetal growth and survival. The significant overlap in FW:PW ratios between the maternal age groups in the human pregnancies is likely reflective of the diversity when studying human pregnancy, emphasising the need for an animal model. The biological significance of a moderate change in placental efficiency in humans (12.2% between controls and mothers ≥40 years) is uncertain; however this is consistent with all women having normal outcomes. The much greater reduction in FW:PW in our mouse model (37.4% in AMA from controls) is likely to reflect the more severe phenotype in the mouse model which presents with high rates of FGR and stillbirth.

Enhanced endothelial-dependent relaxation of uterine arteries was also detected in the AMA mouse model, suggesting that the observations in human pregnancies are not an artefact relating to increased parity and prior irreversible pregnancy-related adaptations in older women^56^. A rat model of AMA found raised systolic blood pressure but reduced uterine artery maximal constriction and no differences in endothelial-dependent vascular relaxation in aged pregnant rats compared to young controls^35^. Comparisons between these models is limited however as their aged dams were calorie constricted and endothelial-independent relaxation of uterine arteries was not reported^35^.

A major difference was the reduced amino acid transport detected in AMA mice, in contrast to the enhanced transport in human pregnancies. This is probably attributable to the key difference in the models. Placentas from human studies were selected to only include those with birthweights in the normal range to exclude any overt effects of pathology on the placenta, whilst mice experienced severe adverse fetal outcomes. The reduced transport in mice corresponded to 56% of pups being growth restricted. Potential mechanisms for the altered transport include down-regulation of placental mTOR or insulin/IGF-I signalling, both of which are known modulators of ageing^57^ and underpin the reduced amino acid transport in a baboon model of FGR^58^. More detailed temporal studies of the mouse model are required to confirm the mechanistic features and to unravel potential adaptive responses. Furthermore, repeating these mouse studies at an intermediate maternal age bracket, such as 24–26 weeks, may result in an intermediate phenotype less severe than the one used in this study.

A limitation of the mouse model is the fact that AMA mice were significantly heavier than control mice. Although this was highly variable, the effect on pregnancy outcome could not be separated from those of ageing. Although obesity is associated with increased adverse outcomes, including macrosomia and stillbirth^59, 60^, it is not associated with reduced litter sizes, severe FGR, or increased placental weight^59, 60^. Furthermore, mouse models of obesity in pregnancy are generated by providing a high fat, obesogenic diet^61, 62^ which may cause adverse outcomes rather than the increased fat mass per se^63^. The higher pre-pregnancy maternal weight in our AMA mice is not due to dietary alterations and restricting their calorie intake would add further confounders^64, 65^. Of note, the pregnancy outcomes for AMA mice with greater maternal weight did not differ from AMA mice without significant age-related maternal weight gain. A further limitation of utilising a mouse model of pregnancy is their polytocous nature, introducing potential confounders, including uterine positioning, litter size and litter-mate competition that do not exist in human pregnancies. However, a carefully chosen animal model can complement human studies providing the limitations are identified and understood.

**Figure 7:**
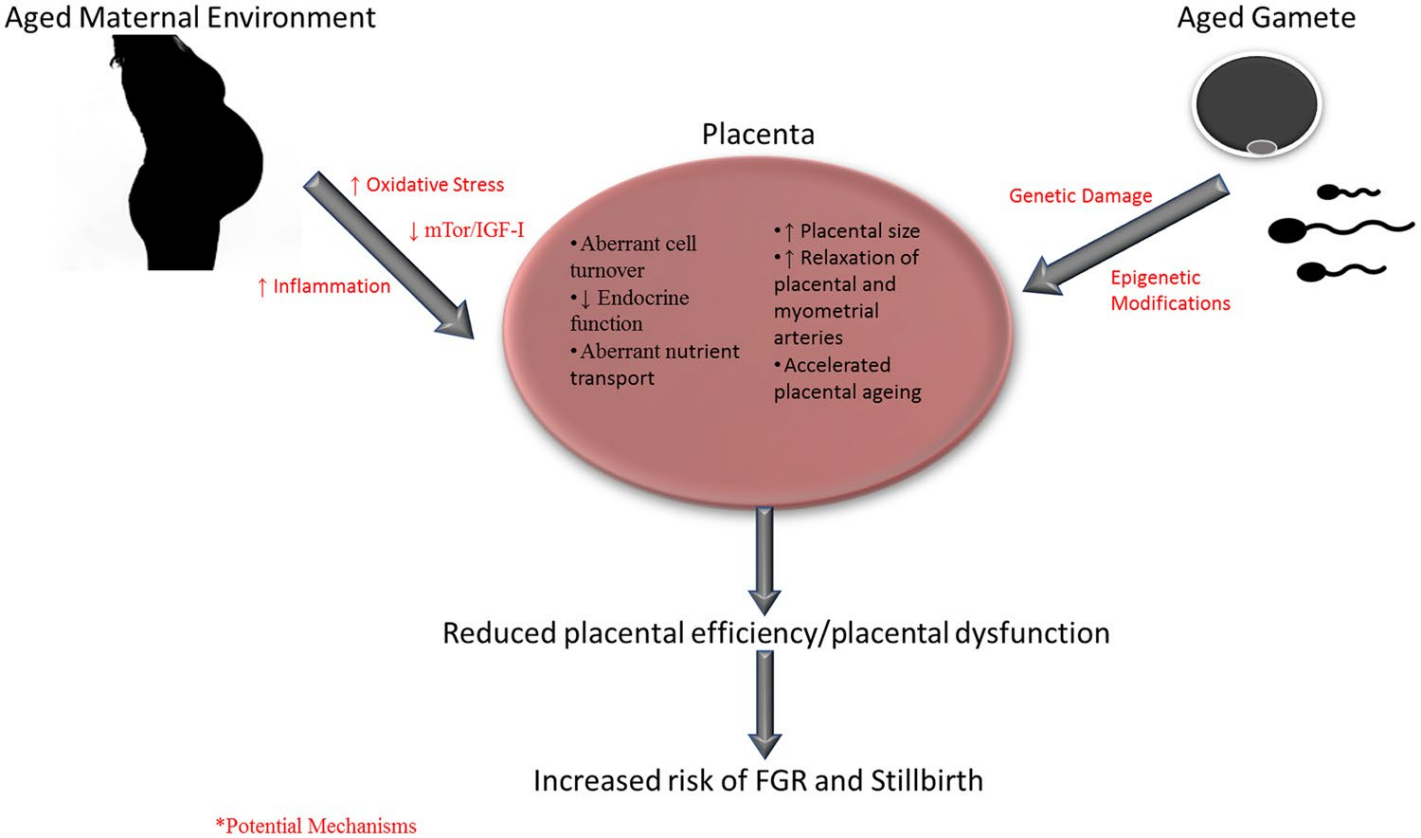
Summary diagram of AMA and placental dysfunction with proposed mechanisms.

The mechanisms underpinning the abnormal placental phenotype in AMA pregnancies remain unknown, but may be related to the ageing oocyte accumulating genetic (and epigenetic) damage^66, 67^ (Fig. 7). Interestingly, our AMA mouse model shares striking similarities to a mouse model of IVF used to investigate placental function and development^68^ including reduced fetal viability and weight and increased fetal loss and placental size, paired with reduced expression and activity of nutrient transport systems. Epigenetic modulation of imprinted genes, namely H19 and PHLDA2, governing early embryo development was cited as the underlying mechanism^68^. The effect of AMA on the epigenetic modulation of imprinted genes is currently unknown^69^. Alternatively, the ageing maternal environment may also contribute to placental dysfunction (Fig. 7). Ageing is associated with increased levels of inflammatory and oxidative stress markers^70^, many of which have been associated with poor pregnancy outcome independent of maternal age^71, 72^. Placental ageing is largely attributed to the placenta being a major source of reactive oxygen species (ROS) which leads to oxidative stress, cellular senescence and loss of tissue function^73, 74^. In *in vitro* models, ROS exposure reproduces some of the placental phenotype seen in AMA pregnancies, such as increased SNAs^22^, altered amino acid transport^46^ and decreased proliferation^75^. A recent study reported increased oxidative stress in the utero-placental bed of aged pregnant mice, and partially improved pregnancy outcome with antioxidant treatment^76^. Further studies are required to determine whether these and other factors in the ageing maternal environment are altered in human pregnancies and are directly related to placental function.

In summary, AMA is associated with multiple aspects of placental dysfunction and altered utero-placental vascular function that are detectable even in pregnancies with apparently normal outcomes. Both human and mouse studies provide evidence for placental and vascular adaptations to support fetal growth, which may be necessary to produce a healthy, adequately grown fetus in an adverse environment. Our human model suggests that placentas from women ≥40 years showed the most severe functional changes and this could guide future studies of AMA. Our mouse model of AMA provides evidence that failure of adaptation leads to severely compromised fetal growth and demise and is a valuable model for delineating the mechanisms causing increased rates of FGR and stillbirth with AMA.

We believe that the evidence presented here is sufficient proof of principal that advancing maternal age affects placental function. The human studies provide evidence of placental dysfunction even in pregnancies that end in apparently normal outcomes, implying underlying and currently unidentified stressors in pregnancy. The mouse model of AMA shows severe placental dysfunction consistent with other studies in FGR and enables analyses of adverse outcomes without the confounders present in human studies. We recognise comparisons between the mouse and human studies should be made cautiously, but emphasise that despite differences in the models, many of the same key markers of placental function are altered. These studies provide the first evidence for a link between advancing maternal age and placental dysfunction and identify important avenues for future study.

## Methods: Human Studies

### Tissue Collection

Women with uncomplicated pregnancies aged 20–30 years (control), 35–39 years, and ≥40 of age years delivering at St Mary’s Hospital, Manchester were recruited to Manchester Advanced Maternal Age Study (MAMAS). Exclusion criteria were BMI <18.5 or ≥30, multiple pregnancy, fetal anomalies and pre-existing (e.g. cardiovascular disease, type I or II diabetes) or current maternal disease that may affect pregnancy (e.g. gestational diabetes mellitus, pre-eclampsia). Data regarding the outcome of pregnancy were recorded from maternal and infant case notes. Only IBCs 10–95 (calculated using GROW software (version 6.7.5.1, UK)) were included. Placentas (from NVDs or caesarean sections; n = 139) and/or myometrium (from caesarean sections; not from placental bed; n = 23) samples were collected (≤1 hour of delivery) after uncomplicated pregnancy. Placentas were weighed and sampled by standard protocols^77^. Placental centiles were calculated based on untrimmed placental weight, gestation and infant sex^78^. Maternal blood was collected following venepuncture (BD Vacutainer CAT) at 28 and 36 weeks gestation and centrifuged to extract serum fractions. Serum samples were stored at −80 °C until analysis.

As it was not possible to use the same placentas for all methodologies, placental function analyses were performed on sub-cohorts of placentas or maternal bloods based on power calculations for each methodology. Demographics were matched where possible to those of the overall study cohort.

### Analysis of Placental Morphology by Histological Techniques

Formalin fixed and paraffin embedded samples from four regions of each placenta (centre, two mid and edge) were used for studies of placental histology (n = 15/age group). Haematoxylin and Eosin staining was performed to assess presence of syncytial nuclear aggregates (SNAs)^79^. Immunohistochemistry using standard colorimetric detection was performed using antibodies for CD31 (Dako, Ely, UK), Ki67 (Dako) and Active Caspase 3 (Abcam, Cambridge UK) as previously described^77^. Ten random images (10x magnification) from each section were taken on an Olympus microscope. SNAs and villous vascularity (based on endothelial CD31 immunostaining) were quantified by manual counting, with normalisation for total villous area using ImagePro (Media Cybernetics). Proliferative and apoptotic indices were calculated following quantification of immunopositive Ki67 and Caspase 3 using HistoQuest (TissueGnostics, Vienna, Austria) for high throughput and unbiased analysis^80^. The number of DAB positive stained nuclei/cells per total number of nuclei detected was determined. All results are displayed as average values of the four regions per placenta.

### Placental Function

#### Placental Amino Acid Transport Activity

Placental amino acid transporter activity was determined by sodium (Na^+^) dependent uptake of ^14^C-methylaminoisbutyric acid (meAIB; System A) and ^3^H-Taurine (TauT) by freshly sampled placental fragments (n = 15/group) as described previously^25, 81^ and analysed as Na^+^-dependent uptake per milligram of villous tissue.

### Circulating Placental Hormones

Concentrations of placental hormones (progesterone, PAPP-A, hPL and hCG (DRG Instruments GmbH, Germany), PlGF and sFlt (VEGF R1/Flt-1) (DuoSet ELISA, R&D Systems Inc. UK) were measured in a subset of maternal serum samples (n = 40/age group, nested case control study design, matched for demographic and biophysical characteristics) collected at 28 and 36 weeks gestation by ELISA by manufacturer’s standard protocol.

### Placental Chorionic Plate Artery Wire Myography

Placental chorionic plate artery (CPA) vascular reactivity was assessed by wire myography as previously described^82^. Arteries (gassed at 5% O_2_) underwent a dose response curve of the thromboxane mimetic U46619 (10^−9^ to 10^−6^ M) to determine maximal constriction of each vessel. From this, an EC_80_ dose of U46619 was applied followed by a dose response of the endothelium independent vasorelaxant sodium nitroprusside (SNP) (10^−11^ to 10^−6^ M). Vascular responses were plotted as averages of the four vessel fragments per placenta and expressed as either change in active effective pressure from baseline (KPa; for constriction) or as a percentage of maximal constriction (for relaxation) (n = 15/age group).

### Myometrial Artery Function by Wire Myography

Myometrial arteries were dissected from myometrial biopsies obtained during Caesarean deliveries (n = 7–14/age group). Arteries (gassed at 18–20% O_2_) were tested for to vasoagonists were determined as above, with the additional assessment of responses to the endothelium dependent vasorelaxant bradykinin (BK; 10^−10^–10^−5^ M). Data were presented as described above.

## Methods: Animal Studies

### Animal Husbandry

C57Bl/6J female mice (Envigo Ltd, United Kingdom) were housed on a 12 hour light-dark cycle at 21–23 °C with free access to food (Beekay Rat and Mouse Diet, Bantin & Kingman, Hull, UK) and water. Our AMA mouse model was based on established murine ageing profiles^55^ and previous studies conducted by Van de Heijden^34^. Control mice (8–16 weeks old) and ageing (38–42 weeks old) mice were mated with C57Bl/6J males (3–6 months). Embryonic day (E) 0.5 of pregnancy was classified at the appearance of a copulation plug. At E17.5, females were sacrificed and fetal and placental tissues were harvested. Late fetal losses were classified as fully formed pups that fail to show signs of life at E17.5 (no detectable movement with one or combinations of: lack of perfusion, early signs of tissue deterioration or negligible detection of radiolabelled ligand post transport studies).

### Fetal and Placental Measurements

Placentas and fetuses were harvested and weights were taken following laparotomy on the dam and expressed as wet weights and as fetal:placental weight ratios as a marker of placental efficiency. Fetal anthropometric measurements were taken by a single observer using thread and a ruler to obtain crown-rump length, abdominal circumference and head circumference measurements as previously described^83^. Fetal and placental weight frequency distribution curves were created as previously described^41^. 2.5^th^, 5^th^ and 95^th^ percentile weights were calculated using the formula: (Z critical value x standard deviation)+ mean with Z critical values of -1.959, -1.649 and 1.649 respectively.

### Unidirectional Maternofetal Transfer of ^14^C-MeAIB or ^3^H-TauT

At E17.5, a subset of dams were anaesthetised with an i.p injection of 1:1:2 mixture of fentanyl/fluanisone (Hypnorm, VetPharma Ltd., Leeds, UK), Midazolam (Roche, UK) and sterile H_2_0 (Braun medical Inc., Pennsylvania, USA) and the tail vein cannulated. The activity of system A transporter was assessed by injection of 140 µl (3.5 µCi) of ^14^C-MeAIB as previously described^84^. This method was optimised for assessment of TauT activity by injection of ^3^H-Taurine (3.5 µCi). Fetuses and placentas were collected within 3 minutes for quantification of radioactivity accumulation in each tissue. Data were presented as K_mf_ (μl/min/g placenta) based on maternal plasma disappearance curves^84^.

### Uterine and Umbilical Artery Function

In a further cohort, culled dams were laparotomised and uterine arteries (UtA) and umbilical arteries (UbA) were dissected. Using wire myography, arteries (gassed at 20% O_2_ for UtA and 5% O_2_ for UbA) were assessed for vasconstriction by exposure to 10^−5^ M phenylephrine (PE, UtA) or U46619 (UbA) for 20 minutes. Vasorelaxation was assessed by dose response of acetylcholine (Ach, 10^−10^–10^−5^ mol/L) or SNP (10^−11^ to 10^−6^ M) to vessels pre-constricted with PE or U46610 (10^−5^ M) respectively. Vascular responses were plotted as averages of the four artery fragments per litter and as a percentage of maximal constriction.

### Statistics

All statistical analyses were performed using GraphPad Prism (Version 6.04, ©1995–2014 GraphPad Software, Inc.). Data was considered significant with *p* values less or equal to 0.05 (95% CI). Statistics performed on continuous data were Mann-Whitney or Kruskal-Wallis if data were not normally distributed or one-way ANOVA and two-way ANOVA as appropriate for normally distributed data with Dunn’s multiple comparisons or Tukey’s post hoc tests where appropriate. Categorical data were analysed by Fishers exact test. Human placental amino acid uptake over time was compared using linear regression. Placental hormones data were log transformed and presented as geometric means with 95% CI and analysed by one-way ANOVA and Tukey’s post hoc test. Mouse data were displayed and statistically analysed as litter averages (N (number of litters), n (number of pups)).

### Study Approval

Written and informed consent was received from all human participants prior to inclusion in this study. All procedures have been approved by NRES Committee North West, (12/NW/0015) and North West REC (08/H1010/55). All methods involving human participants were carried out in accordance with the Human Tissue Act 2004 (HT Act) and accosiated regulations. All animal husbandry and experimental practices were carried out in accordance with the UK Animals (Scientific Procedures) Act 1986 under Home Office Licence 40/3385 and 70/8504. The Local Ethical Review Process of the University of Manchester approved all protocols.

### Data Availability

The datasets generated during and/or analysed during the current study are available from the corresponding author on reasonable request.

## Electronic supplementary material

Supplementary Tables
