# Supplementary material for: Placental Dysfunction Underlies Increased Risk of Fetal Growth Restriction and Stillbirth in Advanced Maternal Age Women
Source: Sci Rep. 2017 Aug 29;7:9677. doi: 10.1038/s41598-017-09814-w (PMC5574918; doi:10.1038/s41598-017-09814-w)
Supplement: Supplementary file 1 — Supplementary Tables [file 41598_2017_9814_MOESM1_ESM.doc]

# Placental Dysfunction Underlies Increased Risk of Fetal Growth Restriction and Stillbirth in Advanced Maternal Age Women

**Authors:** Samantha C. Lean^1*^**,** Alexander E.P Heazell^1,2^, Mark R. Dilworth^1,2^, Tracey A. Mills^1,2^, Rebecca L. Jones^1.2^

**Supplementary Material:**

**Supplementary Table 1: Demographics of participants compared with maternal age for placental histology.**

| **Criteria** | **20-30 years (n=15)** | **35-39 years (n=15)** | **≥40 year**  **(n=15)** | ***p* value** |
| --- | --- | --- | --- | --- |
| **Age** | **27** (20-30) | **37***** (35-39) | **41***** (40-42) | **<0.001***^a^* |
| **BMI**  kg/m^2^ | **25.2**  (18.9-28.8) | **24.9**  (22.829.7) | **22.5**  (20.6-27.2) | NS |
| **Ethnicity**  *Caucasian*  *Other* | **53.3%** (8)  **46.7%** (7) | **73.3%** (11)  **26.7%** (4) | **60.0%** (9)  **40.0%** (6) | NS  NS |
| **Smoking Status**  Smokers | **0%** (0) | **0%** (0) | **0%** (0) | NS |
| **Gravidity** | **1** (1-4) | **2.5** (1-4) | **3*(**1-5) | **0.05** *^a^* |
| **Parity**  *Primiparous*  *Parous* | **53.3%** (8)  **46.7%** (9) | **46.7%** (9)  **53.3%** (8) | **26.7%** (4)  **73.3** (11) | NS |
| **Gestation at Delivery**  Weeks + Days | **40+0**  (38+1 –41+5) | **39+6**  (38+3 –41+5) | **39+3**  (36+0 –41+0) | NS |
| **Mode of Delivery**  *NVD*  *ELCS*  *EMCS* | **60.0%** (9)  **26.7%** (4)  **13.3%** (2) | **46.7%** (9)  **53.3%** (8)  **0%** (0) | **20%*** (3)  **66.7%*** (10)  **13.3%** (2) | **0.05***^b^*  **0.05***^b^*  NS |
| **IBC** | **37.5** (8-88) | **53.5** (12-92) | **52.0** (8-89) | NS |

*Data are median (range) or percentage (number); n=15/age group. Statistical differences are from control group (20-30 Years). ^a^Kruskal-Wallis with Dunn’s multiple comparisons. ^b^Fisher’s exact probability test. BMI = Body Mass Index; NVD = Normal Vaginal Delivery; ELCS = Elective Caesarean Section; EMCS = Emergency Caesarean Section; IBC = Individualised Birthweight Centile.*

Supplementary Table 2: Demographics of participants compared with maternal age for placental transport studies.

| **Demographics** | **20-30 Years**  **(n=15)** | **35-39 Years**  **(n=15)** | **≥40 Years**  **(n=15)** | **Statistics** |
| --- | --- | --- | --- | --- |
| **Maternal Age** Years | **26** (21-29) | **37** (35-39)**** | **43** (40 – 49)** | **<0.0001^a^** |
| **Paternal Age** Years | **20** (21-34) | **37** (24-50)*** | **39** (31-50)*** | **<0.0001^a^** |
| **Ethnicity** *European* | **53.3%** (8) | **86.7%** (13) | **80.0%** (12) | **NS** |
| **BMI** kg/m^2^ | **23.1** (19.0-29.0) | **23.2** (18.7 – 29.2) | **24.7** (20.4-29.1) | NS |
| **Marital Status**  *Married*  *Partner*  *Single* | **60.0%** (9)  **40.0%** (6)  **0.0%** (0) | **73.3%** (11)  **21.4%** (3)  **6.6%** (1) | **53.3%** (8)  **33.3%** (6)  **13.3%** (2) | NS  NS  NS |
| **Employment**  *Employed* | **60.0%** (9) | **80.0%**** (12) | **93.3%***** (14) | **<0.001***^b^* |
| **Non-Smokers** | **93.3%** (14) | **100%** (15) | **100%** (15) | NS |
| **Housing Status**  *Owns* | **26.6%** (4) | **73.3%** (11) | **80.0**%*(12) | **<0.01^a^** |
| **Parity**  *Primips*  *Parous*  *Grandparous* | **40.0%** (6)  **73.3%** (11)  **0.0%** (0) | **40.0%** (6)  **60.0%** (9)  **0.0%** (0) | **40.0%** (6)  **60.0%** (9)  **0.0%** (0) | NS  NS  NS |
| **Previous Miscarriage** | **7.1%** | **14.2%** | **40.0%**** | **<0.05^b^** |
| **Previous APO**  *Of parous women* | **0.0%** (0)  **0.0%** (0) | **6.7%** (1)  **11.1%** (1) | **6.7%** (1)  **11.1%** (1) | NS  NS |
| **Fertility Treatment**  *Previous*  *Current* | **0.0%** (0)  **0.0%** (0) | **6.7%** (1)  **13.3%** (2) | **13.3%** (2)  **20.0%** (3) | NS  NS |
| **Gestation at Delivery** | **39+5**  (37+2 –41+6) | **40+0**  ( 38+4- 42+2) | **39+3**  (38+0 –41+0) | NS |
| **Birthweight**  g | **3311**  (2620-4300) | **3429**  (3142 –4100) | **3629**  (3014 – 4420) | NS |
| **IBC** | **39.6**  (10.1-87.9) | **47.0**  (16.9-81.1) | **65.8**  (21.4 – 99.4)* | **<0.05***^a^* |
| **Induction Rate** | **35.7%** | **21.2%** | **26.7%** | NS |
| **Mode of Delivery**  *NVD*  *ELCS*  *EMCS*  *INST* | **60.0%** (9)  **33.3%** (5)  **0.0%** (0)  **6.7%** (1) | **33.3%** (5)  **33.3%** (5)  **6.7%** (1)  **26.6%** (4) | **40.0%** (6)  **40.0%** (6)  **13.3%** (2)  **6.7%** (1) | NS  NS  NS  NS |
| **Male Infant** | **53.3%** (8) | **80.0%** (12) | **46.7%** (7) | NS |

*Data are median (range) or percentage (number); n=15/age group. Statistical differences are from control group (20-30 Years). ^a^Kruskal-Wallis with Dunn’s multiple comparisons. ^b^Fisher’s exact probability test. BMI = Body mass Index; APO = Adverse Pregnancy Outcome; IBC = Individualised Birthweight Centile; NVD = Normal Vaginal Delivery; ELCS = Elective Caesarean Section; EMCS = Emergency Caesarean Section; INST = Instrumental Delivery.*
